# Chronic Metformin Therapy is Associated with a Lower Risk of Hemorrhoid in Patients with Type 2 Diabetes Mellitus

**DOI:** 10.3389/fphar.2020.578831

**Published:** 2021-02-16

**Authors:** Chin-Hsiao Tseng

**Affiliations:** ^1^ Department of Internal Medicine, National Taiwan University College of Medicine, Taipei, Taiwan; ^2^ Division of Endocrinology and Metabolism, Department of Internal Medicine, National Taiwan University Hospital, Taipei, Taiwan; ^3^ Division of Environmental Health and Occupational Medicine of the National Health Research Institutes, Zhunan, Taiwan

**Keywords:** diabetes mellitus, hemorrhoid, metformin, National Health Insurance, pharmacoepidemiology, Taiwan

## Abstract

**Background:** Metformin has anti-inflammatory property and reduces the risk of varicose vein in our previous study.

**Aim:** To investigate the risk of hemorrhoid, another common disease involving the hemorrhoidal venous plexus, in ever vs. never users of metformin in patients with type 2 diabetes mellitus.

**Methods:** This is a population-based retrospective cohort study. Patients with new-onset type 2 diabetes mellitus during 1999–2005 were enrolled from Taiwan’s National Health Insurance. All patients who were alive on January 1, 2006 were followed up until December 31, 2011. Analyses were conducted in both an unmatched cohort of 152,347 ever users and 19,523 never users and in 19,498 propensity score (PS)-matched pairs of ever and never users. Traditional Cox regression and Cox regression incorporated with the inverse probability of treatment weighting (IPTW) using the PS were used to estimate hazard ratios.

**Results:** New-onset hemorrhoid was diagnosed in 8,211 ever users and 2025 never users in the unmatched cohort and in 1,089 ever users and 2022 never users in the matched cohort. The hazard ratio for ever vs. never users derived from the traditional Cox regression was 0.464 (95% confidence interval: 0.440–0.488) in the unmatched cohort; and was 0.488 (0.453–0.525) in the matched cohort. In the IPTW models, the hazard ratio was 0.464 (0.442–0.487) in the unmatched cohort and was 0.492 (0.457–0.530) in the matched cohort. A dose-response pattern was observed while comparing the tertiles of cumulative duration, cumulative dose and defined daily dose of metformin therapy to never users in all analyses. A risk reduction of approximately 40–50% was consistently observed in various sensitivity analyses.

**Conclusion:** Chronic therapy with metformin in patients with type 2 diabetes mellitus is associated with a lower risk of hemorrhoid.

## Introduction

Hemorrhoid is a very common disease that affects the anorectal area resulting in distal displacement of the anal cushions. Clinical presentations include vascular congestion, inflammation, itching, soiling, pain, bleeding and prolapse. The precise cause remains unknown but conditions that increase intra-abdominal pressure may increase the pressure in the hemorrhoidal venous plexus and precipitate its development. These include straining during constipation, chronic diarrhea, irritable bowel syndrome, pregnancy, delivery, obesity, lack of exercise, low-fiber diets, cigarette smoking, anal intercourse, long-time standing, cirrhosis with ascites, pelvic floor dysfunction and chronic cough (Johannsson et al., 2005; Helvaci et al., 2009; Mott et al., 2018; Yetkin and Ozturk, 2018; Ekici et al., 2019; Nagaraj et al., 2019). Hemorrhoid is age-related with peak prevalence at the age of 45–65 years. Its prevalence was 39% in a routine colorectal cancer screening conducted in Vienna, Austria and among them 55% were asymptomatic (Riss et al., 2012). Medical management, dietary modification and behavioral therapies are initial treatment, but surgical interventions may be necessary in some patients (Mott et al., 2018).

Hemorrhoid and varicose veins share common pathophysiology and may have similar risk factors (Mott et al., 2018; Yetkin and Ozturk, 2018; Ekici et al., 2019). Metformin is now recommended by major treatment guidelines as the first-line therapy for patients with type 2 diabetes mellitus (American Diabetes Association, 2017; Salvatore et al., 2020) because of its multiple benefits beyond glycemic control, including immune modification, anti-inflammation, anti-atherosclerosis, anti-cancer and anti-aging (Wang et al., 2017). Our recent study suggested that use of metformin in patients with type 2 diabetes mellitus is also associated with a lower risk of varicose veins (Tseng, 2020). To our knowledge, there has been no previous study investigating whether metformin use might reduce the risk of hemorrhoid. Because it is reasonable to speculate that the beneficial effect of metformin on varicose veins might also be applied to hemorrhoid, the purpose of the present study was to evaluate whether metformin use in patients with type 2 diabetes mellitus could be associated with a lower risk of hemorrhoid, by using a nationwide reimbursement database of the Taiwan’s National Health Insurance (NHI) and comparing the risk of hemorrhoid between ever users and never users of metformin, in both an unmatched cohort and a cohort of 1:1 matched pairs of ever and never users who were well balanced in characteristics based on propensity score (PS).

## Materials and Methods

Taiwan has implemented a universal and compulsory healthcare system, the NHI, since March 1995. More than 99% of Taiwan’s population is covered by the NHI. The Bureau of the NHI has contracts with all hospitals and 93% of all medical settings, and keeps all computer records of disease diagnoses, medication prescriptions and clinical procedures submitted for reimbursement purpose. After ethics review and approval by the Research Ethics Committee of the National Health Research Institutes, the reimbursement database can be used for academic research. Informed consent from the patients was not required according to the local regulations because all personal information has been de-identified for the protection of privacy. The present study was granted an approval number of 99274.

Disease diagnoses during the study period were coded by the International Classification of Diseases, Ninth Revision, Clinical Modification (ICD-9-CM). Diabetes was coded 250.XX and hemorrhoid was coded 455.

**FIGURE 1:**
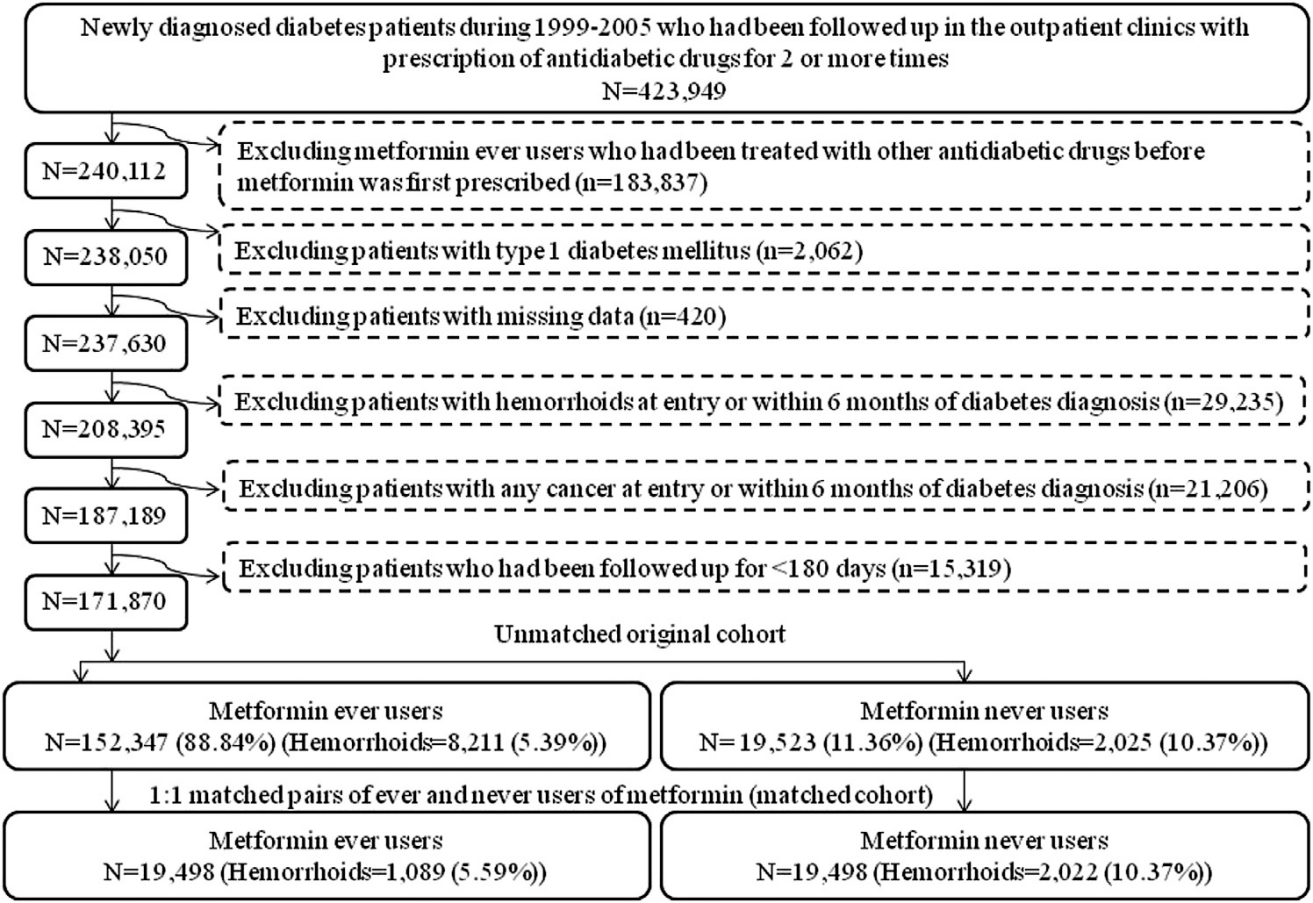
Flowchart showing the procedures in creating an unmatched cohort and a cohort of 1:1 matched-pairs (based on propensity score) of metformin ever users and never users from the reimbursement database of the Taiwan’s National Health Insurance.

The database has been described in more detail in a previously published paper (Tseng, 2017). The procedures used to enroll an unmatched original cohort and a cohort of 1:1 PS-matched pairs of ever and never users of metformin derived from the original cohort are shown in Figure 1. At first, we identified 423,949 patients with a new diagnosis of diabetes mellitus during 1999–2005 in the outpatient clinics and having been prescribed antidiabetic drugs for 2 or more times in the database. The following ineligible patients were then excluded: 1) ever users of metformin who had been prescribed other antidiabetic drugs before the initiation of metformin (*n* = 183,837); 2) patients with a diagnosis of type 1 diabetes mellitus (*n* = 2,062), 3) patients with missing data (*n* = 420), 4) patients with a diagnosis of hemorrhoid before entry or within 6 months of the diagnosis of diabetes mellitus (*n* = 29,235), 5) patients with a diagnosis of any cancer before entry or within 6 months of the diagnosis of diabetes mellitus (*n* = 21,206), 6) patients who had been followed up for <180 days (*n* = 15,319). As a result, 152,347 ever users of metformin and 19,523 never users of metformin were identified and they were considered as the unmatched original cohort. All characteristics shown in Table 1 plus the date of entry were then used to create the PS by logistic regression. A cohort of 19,498 PS-matched pairs of ever users and never users of metformin (the matched cohort) was then created from the unmatched cohort by using the Greedy 8→1 digit match algorithm proposed by Parsons (Parsons, 2020). The following analyses were conducted in both the unmatched cohort and the matched cohort to examine the consistency of the findings.

**TABLE 1:** Characteristics in never and ever users of metformin in the unmatched cohort and the matched cohort.

Variable	Unmatched cohort					Matched cohort				
	Never users (*n* = 19,523)		Ever users (*n* = 152,347)		SD	Never users (*n* = 19,498)		Ever users (*n* = 19,498)		SD
	*n*	%	*n*	%		*n*	%	*n*	%	
Demographic data										
Age^a^ (years)	68.32	13.34	64.11	11.94	−39.37	68.30	13.33	68.09	12.29	−1.09
Sex (men)	10,548	54.03	79,205	51.99	−4.11	10,537	54.04	10,430	53.49	−1.28
Occupation										
I	7,039	36.05	56,816	37.29		7,032	36.07	7,010	35.95	
II	3,249	16.64	32,363	21.24	13.52	3,246	16.65	3,273	16.79	0.25
III	4,814	24.66	35,482	23.29	−3.51	4,809	24.66	4,885	25.05	1.21
IV	4,421	22.65	27,686	18.17	−13.11	4,411	22.62	4,330	22.21	−0.89
Living region										
Taipei	6,518	33.39	47,352	31.08		6,508	33.38	6,404	32.84	
Northern	2,061	10.56	17,604	11.56	3.38	2,061	10.57	2,057	10.55	−0.04
Central	3,359	17.21	27,594	18.11	2.21	3,353	17.20	3,391	17.39	0.49
Southern	3,438	17.61	26,418	17.34	−0.56	3,435	17.62	3,447	17.68	0.40
Kao-Ping and Eastern	4,147	21.24	33,379	21.91	2.61	4,141	21.24	4,199	21.54	0.86
Major comorbidities										
Hypertension	16,513	84.58	126,762	83.21	−5.14	16,489	84.57	16,474	84.49	−0.03
Dyslipidemia	13,461	68.95	123,784	81.25	33.25	13,452	68.99	13,535	69.42	0.92
Obesity	452	2.32	6,212	4.08	10.69	452	2.32	479	2.46	0.90
Diabetes-related complications										
Nephropathy	7,173	36.74	43,358	28.46	−22.29	7,153	36.69	7,083	36.33	−1.15
Eye diseases	3,284	16.82	48,021	31.52	36.51	3,283	16.84	3,140	16.10	−2.60
Diabetic polyneuropathy	3,302	16.91	44,227	29.03	30.82	3,302	16.94	3,272	16.78	−0.78
Stroke	7,710	39.49	49,996	32.82	−18.19	7,689	39.43	7,562	38.78	−1.22
Ischemic heart disease	10,191	52.20	72,530	47.61	−11.73	10,175	52.18	10,001	51.29	−1.62
Peripheral arterial disease	4,864	24.91	40,714	26.72	2.95	4,854	24.89	4,733	24.27	−1.59
Antidiabetic drugs										
Insulin	1,658	8.49	3,487	2.29	−34.88	1,641	8.42	1,489	7.64	−5.15
Sulfonylurea	14,157	72.51	109,708	72.01	8.27	14,155	72.60	14,649	75.13	4.77
Meglitinide	1,695	8.68	6,113	4.01	−21.47	1,689	8.66	1,681	8.62	−0.56
Acarbose	2,138	10.95	8,189	5.38	−19.59	2,131	10.93	2,301	11.80	0.79
Rosiglitazone	558	2.86	7,270	4.77	11.29	558	2.86	613	3.14	0.72
Pioglitazone	444	2.27	3,866	2.54	3.24	443	2.27	473	2.43	0.17
Commonly encountered comorbidities and potential risk factors										
Chronic obstructive pulmonary disease	10,476	53.66	76,095	49.95	−10.34	10,454	53.62	10,478	53.74	0.45
Tobacco abuse	440	2.25	5,437	3.57	8.61	440	2.26	394	2.02	−1.70
Alcohol-related diagnoses	1,188	6.09	9,438	6.20	0.39	1,188	6.09	1,220	6.26	0.55
Cancer	1,822	9.33	11,224	7.37	−7.67	1,817	9.32	1,872	9.60	0.94
Heart failure	5,462	27.98	30,250	19.86	−24.05	5,449	27.95	5,267	27.01	−2.03
Parkinson’s Disease	1,120	5.74	5,625	3.69	−12.10	1,112	5.70	1,047	5.37	−1.30
Dementia	2,196	11.25	10,980	7.21	−19.61	2,508	12.86	2,364	12.12	−2.05
Head injury	730	3.74	6,034	3.96	1.18	729	3.74	691	3.54	−1.09
Valvular heart disease	2,791	14.30	15,552	10.21	−16.13	2,785	14.28	2,707	13.88	−1.05
Commonly used medications in diabetes patients										
Angiotensin converting enzyme inhibitors/angiotensin receptor blockers	14,238	72.93	113,939	74.79	3.61	14,216	72.91	14,178	72.72	−0.38
Calcium channel blockers	13,118	67.19	94,952	62.33	−12.16	13,099	67.18	13,036	66.86	−0.43
Statins	10,002	51.23	98,120	64.41	30.28	9,999	51.28	9,958	51.07	−0.66
Fibrates	6,239	31.96	63,635	41.77	23.11	6,237	31.99	6,161	31.60	−0.84
Aspirin	11,985	61.39	96,528	63.36	2.65	11,966	61.37	12,019	61.64	0.70

^a^ Age is expressed as mean and standard deviation.

Refer to “Materials and Methods” for the classification of occupation.

SD: standardized difference.

Potential confounders were categorized into the following subgroups: demographic data, major comorbidities, diabetes-related complications, antidiabetic drugs, commonly encountered comorbidities and potential risk factors and commonly used medications in patients with diabetes mellitus. Demographic data included age, sex, occupation and living region. Major comorbidities included in the analyses were hypertension (401–405), dyslipidemia (272.0–272.4) and obesity (278). Diabetes-related complications included nephropathy (580–589), eye diseases (250.5: diabetes with ophthalmic manifestations, 362.0: diabetic retinopathy, 369: blindness and low vision, 366.41: diabetic cataract, and 365.44: glaucoma associated with systemic syndromes), diabetic polyneuropathy (357.2 and 250.6), stroke (430–438), ischemic heart disease (410–414) and peripheral arterial disease (250.7, 785.4, 443.81 and 440–448). Antidiabetic drugs were categorized as insulin, sulfonylurea, meglitinide, acarbose, rosiglitazone and pioglitazone. Commonly encountered comorbidities and potential risk factors of hemorrhoid included chronic obstructive pulmonary disease (a surrogate for smoking, 490–496), tobacco abuse (305.1, 649.0 and 989.84), alcohol-related diagnoses (291, 303, 535.3, 571.0–571.3 and 980.0), cancer (140–208), heart failure (398.91, 402.11, 402.91, 404.11, 404.13, 404.91, 404.93 and 428), Parkinson’s disease (332), dementia (abridged codes of A210 or A222, or ICD-9-CM codes of 290.0, 290.1, 290.2, 290.4, 294.1, 331.0–331.2 and 331.7–331.9), head injury (959.01) and valvular heart disease (394–396, 424 and 746). Medications commonly used by patients with diabetes mellitus included angiotensin-converting enzyme inhibitors/angiotensin receptor blockers, calcium channel blockers, statins, fibrates and aspirin.

The living region and occupation were described in detail elsewhere (Tseng, 2012). In brief, the living region was classified as Taipei, Northern, Central, Southern, and Kao-Ping/Eastern. Occupation was classified as class I (civil servants, teachers, employees of governmental or private businesses, professionals and technicians), class II (people without a specific employer, self-employed people or seamen), class III (farmers or fishermen) and class IV (low-income families supported by social welfare, or veterans).

Standardized difference for each of the above potential confounders was calculated as a test of balance diagnostic according to Austin and Stuart (2015). A cutoff value of >10% was used as an indication of potential confounding from the variable.

Cumulative duration of metformin therapy in months, cumulative dose of metformin therapy in grams and units of defined daily dose (DDD) of metformin use per day [1 unit of DDD for metformin = 2 g (Chang et al., 2018)] were calculated and their tertiles were used for dose-response analyses. Incidence density of hemorrhoid was calculated for never users of metformin, ever users of metformin, the tertiles of cumulative duration of metformin therapy, the tertiles of cumulative dose of metformin therapy and the tertiles of units of DDD of metformin use per day. Start of follow-up was set on January 1, 2006. The numerator of the incidence was the case number of newly identified hemorrhoid during follow-up. The denominator (expressed in person-years) was the follow-up duration between the start of follow-up and the time of a new diagnosis of hemorrhoid, or the date of death or the date of the last reimbursement record, whichever occurred first up to December 31, 2011.

Kaplan–Meier curves of hemorrhoid-free probability were plotted for never users and ever users of metformin, for never users and tertiles of cumulative duration of metformin therapy, for never users and tertiles of cumulative dose of metformin therapy and for never users and tertiles of units of DDD per day. The significance in different subgroups of metformin exposure was tested by logrank test.

The subgroup of never users of metformin was used as the referent group in the estimation of hazard ratios and their 95% confidence intervals for hemorrhoid for ever users and for each tertile of cumulative duration, cumulative dose and units of DDD. Both traditional Cox regression and Cox regression incorporated with the inverse probability of treatment weighting (IPTW) using the PS were used to examine the consistency of the findings. The IPTW method was proposed by Austin to reduce the potential confounding from the differences in characteristics (Austin, 2013).

Sensitivity analyses were conducted by estimating the overall hazard ratios for ever users vs. never users in more homogeneous subgroups of patients. First, patients with irregular refill of metformin were excluded. This was done by excluding patients who received two consecutive prescriptions of metformin spanning a period of >4 months (Model I). Because the NHI allows drug prescription for chronic diseases for not more than 3 months at each time, these patients represented those who have delayed refill of metformin for more than one month after a previous prescription for 3 months. Second, patients who happened to be treated with incretin-based therapies, either with a dipeptidyl peptidase 4 inhibitor or a glucagon-like peptide 1 receptor agonist, during the follow-up period were excluded (Model II). In Taiwan, the first incretin-based therapy was not reimbursed by the NHI until after 2009. The exclusion of these patients avoided the potential impact of incretin-based therapies during follow-up. Third, patients enrolled during two different periods of 1999–2002 (Model III) and 2003–2005 (Model IV) were analyzed separately. Because more and more antidiabetic drugs have been introduced into clinical use and the guidelines for the use of antidiabetic drugs have evolved over the last 2 decades, these sensitivity analyses examined whether the results could be influenced by these changes. Fourth, to reduce the potential risk of misdiagnosis and misclassification of hemorrhoid at the outpatient clinics, analysis was performed by re-defining the outcome of hemorrhoid by using a more stringent criteria, i.e., as a primary diagnosis at hospitalization (Model V). These hospitalized patients might represent those who had more severe clinical manifestations of hemorrhoid and surgical intervention or more intensive medical care was required. Fifth, subgroup analyses were conducted with regards to the use of aspirin (Model VI: patients receiving aspirin; Model VII: patients not receiving aspirin) and calcium channel blockers (Model VIII: patients receiving calcium channel blockers; Model IX: patients not receiving calcium channel blockers) because these medications may potentially cause bias relating to disease diagnosis. Aspirin can increase the risk of hemorrhoidal bleeding (Davis et al., 2018) but on the other hand it may also be used for hemorrhoidal pain relief (Sun and Migaly, 2016). Calcium channel blockers can reduce resting anal pressure and have been used for the treatment of hemorrhoid (Lohsiriwat, 2012) and anal fissure (Sahebally et al., 2017).

Analyses were conducted using SAS statistical software, version 9.4 (SAS Institute, Cary, NC). *p* < 0.05 was considered statistically significant.

## Results

The characteristics in never users and ever users of metformin in the unmatched cohort and the matched cohort, respectively, are shown in Table 1. Many of the covariates were not balanced between never and ever users of metformin as indicated by a standardized difference >10% in the unmatched cohort. However, all covariates were well balanced between the two groups in the matched cohort because none of them had a value of standardized difference >10%.

**FIGURE 2:**
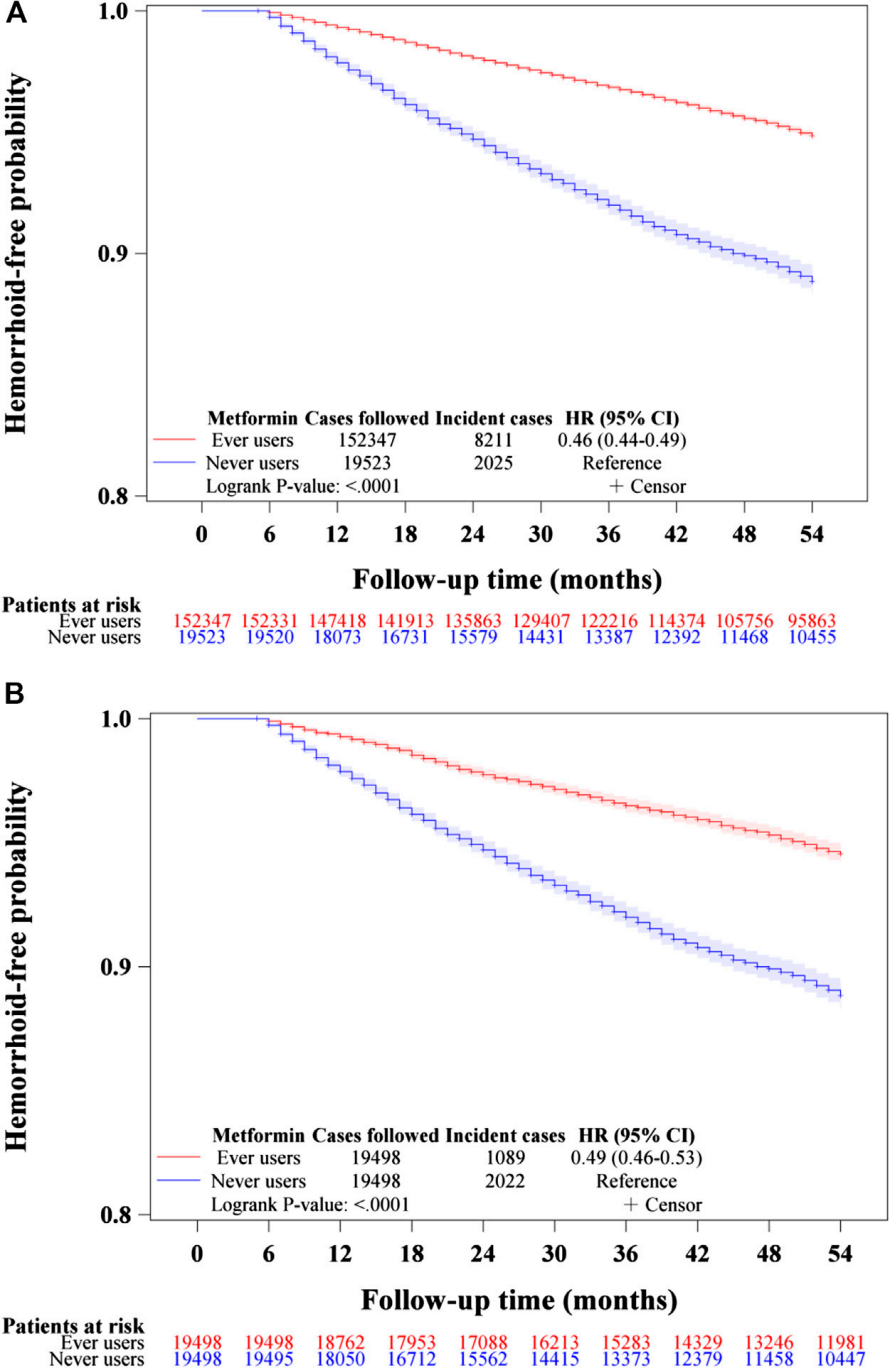
Kaplan–Meier curves comparing hemorrhoid-free probability in never users and ever users of metformin in the unmatched cohort **(A)** and the matched cohort **(B)**. HR: hazard ratio, CI: confidence interval.

The Kaplan-Meier curves comparing hemorrhoid-free probability with regards to metformin exposure are shown in Figure 2. Figure 2A shows the curves for never users and ever users in the unmatched cohort and Figure 2B shows the respective curves in the matched cohort. The *p*-values of the logrank test were <0.0001 in both the unmatched cohort and the matched cohort.

**FIGURE 3:**
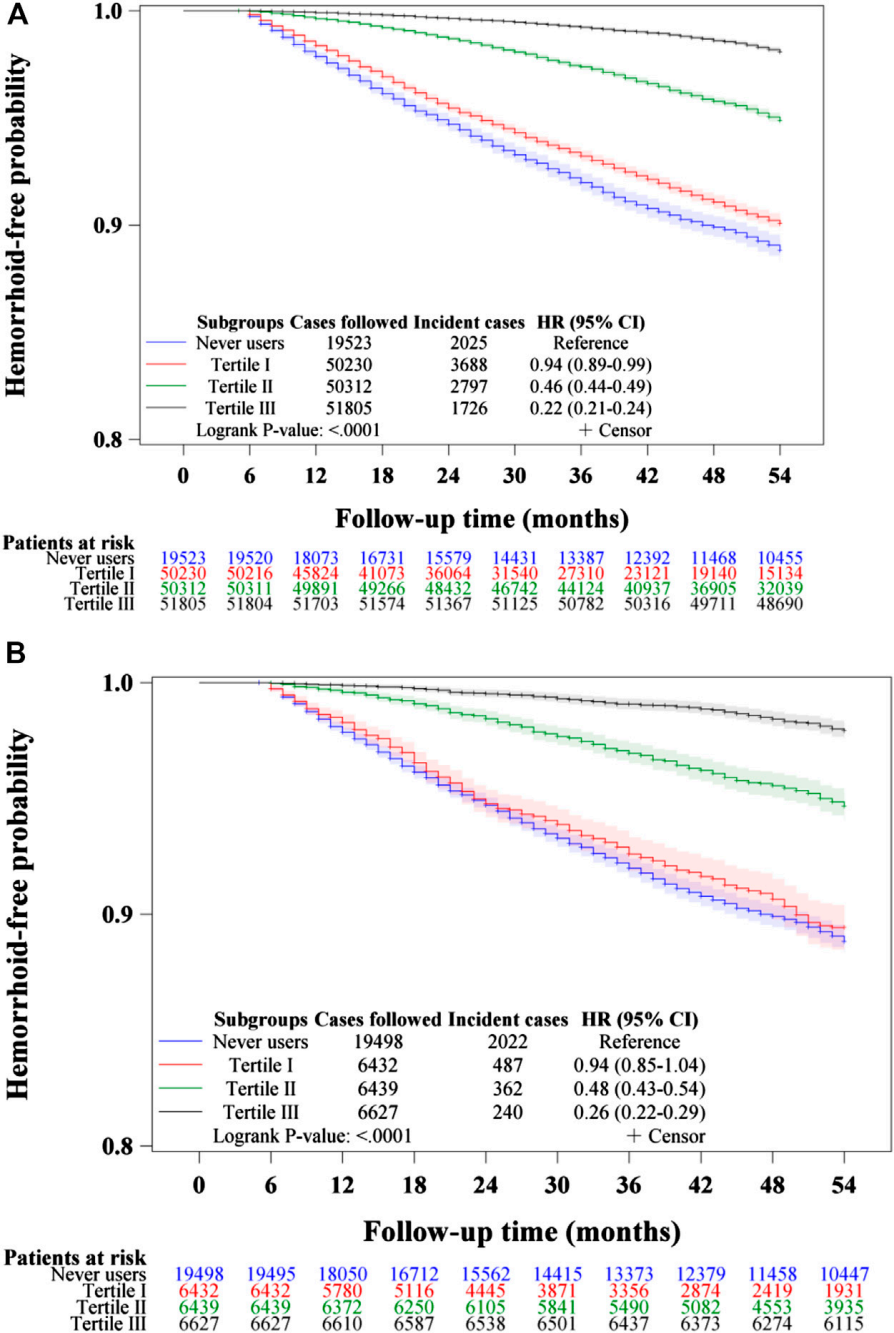
Kaplan–Meier curves comparing hemorrhoid-free probability among never users and tertiles of cumulative duration of metformin therapy in the unmatched cohort **(A)** and the matched cohort **(B)**, respectively. HR: hazard ratio, CI: confidence interval.

**FIGURE 4:**
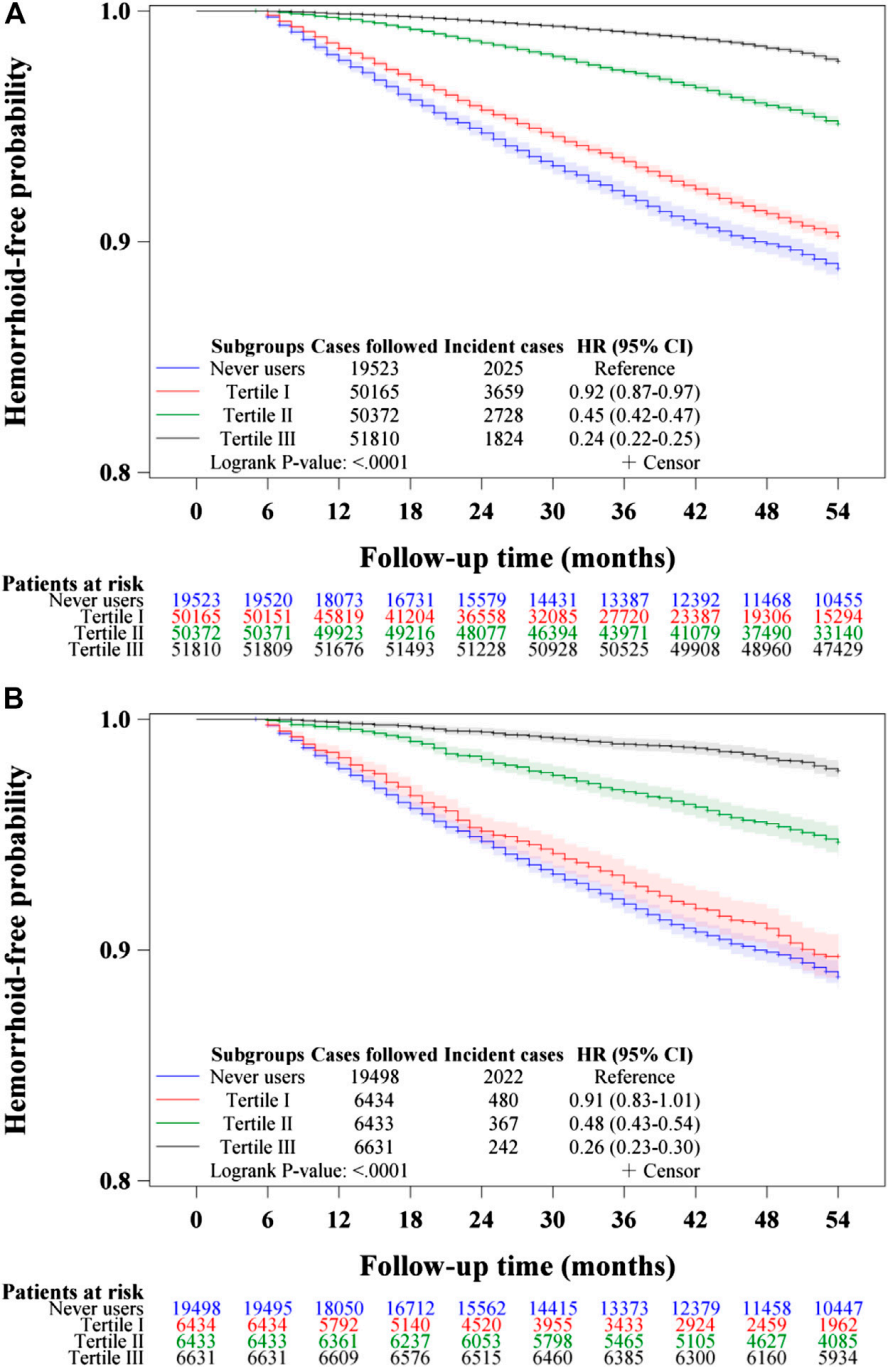
Kaplan–Meier curves comparing hemorrhoid-free probability among never users and tertiles of cumulative dose of metformin therapy in the unmatched cohort **(A)** and the matched cohort **(B)**, respectively.

**FIGURE 5:**
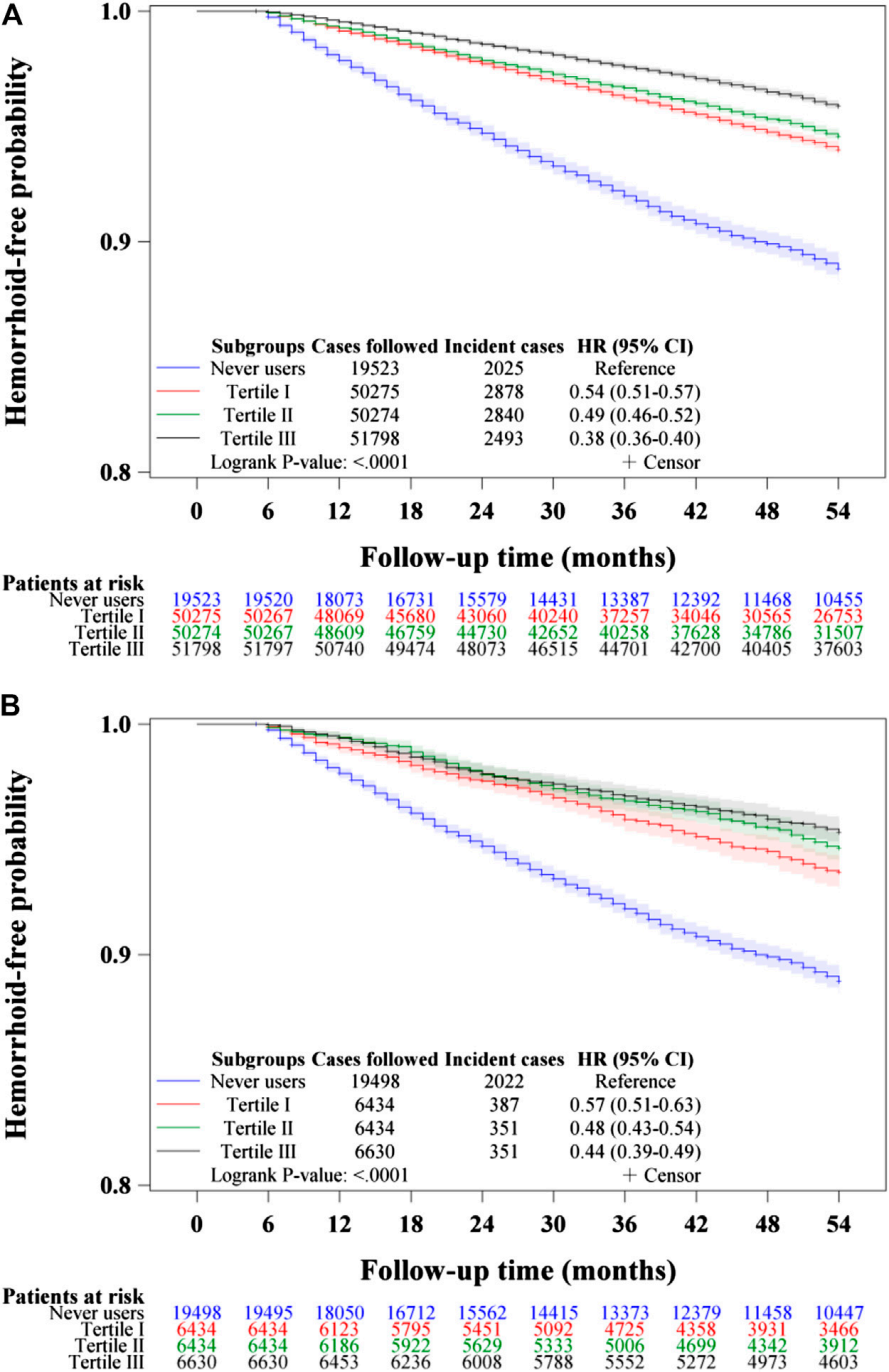
Kaplan–Meier curves comparing hemorrhoid-free probability among never users and tertiles of units of defined daily dose of metformin in the unmatched cohort **(A)** and the matched cohort **(B)**, respectively.

Figures 3–5 show the Kaplan-Meier curves comparing hemorrhoid-free probability in never users of metformin and in ever users of metformin categorized according to the tertiles of cumulative duration of metformin therapy (Figure 3), the tertiles of cumulative dose of metformin therapy (Figure 4) and the tertiles of units of DDD per day (Figure 5), respectively. Figures 3A, 4A, and 5A show the curves in the unmatched cohort; and Figures 3B, 4B, and 5B show the respective curves in the matched cohort. The logrank test (*p* < 0.0001) supported a significant difference in a dose-response pattern among the various subgroups of metformin exposure in all three parameters.

Table 2 shows the incidence of hemorrhoid and the hazard ratios by metformin exposure in the unmatched cohort and the matched cohort, respectively. A significantly lower risk in ever users could be demonstrated by the overall hazard ratios in both the traditional Cox regression and the Cox regression incorporated with IPTW in either the unmatched cohort or the matched cohort. A dose-response relationship could be seen in the tertile analyses in all models. Significant *p*-values were noted for metformin use for more than approximately 2 years in the cumulative duration analyses (in the second and third tertiles); for more than approximately 750 grams in the cumulative dose analyses (in the second and third tertiles); and for all tertiles in the units of DDD per day analyses. Analyses in the tertiles of units of DDD suggested that the protective effect could be seen across all tertiles with a trend of greater protection in higher daily dose. In the unmatched cohort, the mean (median) values of cumulative duration, cumulative dose and units of DDD of metformin therapy among ever users were 45.7 (40.6) years, 1,692.7 (1,300.0) grams and 0.58 (0.54) units of DDD, respectively. In the matched cohort, the respective values were 45.0 (40.1) years, 1,650.1 (1,265.7) grams and 0.57 (0.54) units of DDD.

**TABLE 2:** Incidence rates of hemorrhoid and hazard ratios by metformin exposure.

Model/Metformin use	IncidentCase number	CasesFollowed	Person-years	Incidence rate(per 100,000 person-years)	Traditional Cox model			IPTW model		
					HR	95% CI	p Value	HR	95% CI	p Value
Unmatched cohort										
Never users	2,025	19,523	80,153.12	2,526.41	1.000			1.000		
Ever users	8,211	152,347	692,486.07	1,185.73	0.464	(0.440–0.488)	<0.0001	0.464	(0.442–0.487)	<0.0001
Tertiles of cumulative duration of metformin therapy (months)										
Never users	2,025	19,523	80,153.12	2,526.41	1.000			1.000		
<25.5	3,688	50,230	165,167.79	2,232.88	1.038	(0.978–1.101)	0.2170	0.874	(0.827–0.923)	<0.0001
25.5–56.7	2,797	50,312	236,249.97	1,183.92	0.477	(0.449–0.506)	<0.0001	0.459	(0.434–0.486)	<0.0001
>56.7	1,726	51,805	291,068.31	592.99	0.217	(0.203–0.232)	<0.0001	0.218	(0.205–0.233)	<0.0001
Tertiles of cumulative dose of metformin therapy (grams)										
Never users	2,025	19,523	80,153.12	2,526.41	1.000			1.000		
<756	3,659	50,165	166,490.45	2,197.72	1.019	(0.960–1.081)	0.5398	0.864	(0.818–0.912)	<0.0001
756–1960	2,728	50,372	238,241.26	1,145.06	0.455	(0.429–0.484)	<0.0001	0.444	(0.420–0.471)	<0.0001
>1960	1,824	51,810	287,754.36	633.87	0.231	(0.216–0.246)	<0.0001	0.235	(0.221–0.251)	<0.0001
Tertiles of units of defined daily dose of metformin therapy per day										
Never users	2,025	19,523	80,153.12	2,526.41	1.000			1.000		
<0.49	2,878	50,275	211,323.75	1,361.89	0.531	(0.500–0.564)	<0.0001	0.536	(0.507–0.568)	<0.0001
0.49–0.65	2,840	50,274	227,907.44	1,246.12	0.488	(0.459–0.518)	<0.0001	0.489	(0.462–0.517)	<0.0001
>0.65	2,493	51,798	253,254.89	984.38	0.387	(0.364–0.411)	<0.0001	0.382	(0.360–0.405)	<0.0001
Matched cohort										
Never users	2,022	19,498	80,068.10	2,525.35	1.000			1.000		
Ever users	1,089	19,498	87,196.70	1,248.90	0.488	(0.453–0.525)	<0.0001	0.492	(0.457–0.530)	<0.0001
Tertiles of cumulative duration of metformin therapy (months)										
Never users	2,022	19,498	80,068.10	2,525.35	1.000			1.000		
<24.9	487	6,432	20,745.05	2,347.55	0.985	(0.888–1.092)	0.7740	0.915	(0.828–1.011)	0.0799
24.9–56.0	362	6,439	29,625.10	1,221.94	0.481	(0.430–0.538)	<0.0001	0.478	(0.427–0.534)	<0.0001
>56.0	240	6,627	36,826.55	651.70	0.245	(0.214–0.280)	<0.0001	0.256	(0.224–0.293)	<0.0001
Tertiles of cumulative dose of metformin therapy (grams)										
Never users	2,022	19,498	80,068.10	2,525.35	1.000			1.000		
<736	480	6,434	20,985.04	2,287.34	0.975	(0.879–1.083)	0.6395	0.893	(0.808–0.987)	0.0260
736–1918	367	6,433	29,821.41	1,230.66	0.483	(0.432–0.540)	<0.0001	0.482	(0.431–0.539)	<0.0001
>1918	242	6,631	36,390.26	665.01	0.247	(0.216–0.283)	<0.0001	0.262	(0.229–0.299)	<0.0001
Tertiles of units of defined daily dose of metformin therapy per day										
Never users	2,022	19,498	80,068.10	2,525.35	1.000			1.000		
<0.49	387	6,434	26,985.17	1,434.12	0.574	(0.514–0.642)	<0.0001	0.564	(0.506–0.629)	<0.0001
0.49–0.64	351	6,434	28,633.36	1,225.84	0.476	(0.425–0.533)	<0.0001	0.483	(0.431–0.541)	<0.0001
>0.64	351	6,630	31,578.18	1,111.53	0.427	(0.381–0.479)	<0.0001	0.439	(0.392–0.492)	<0.0001

Hemorrhoid was based on a diagnosis made at the out-patient clinics or during hospitalization.

^a^ Unit of defined daily dose of metformin = 2 grams.

IPTW: inverse probability of treatment weighting, HR: hazard ratio, CI: confidence interval.

The sensitivity analyses shown in Table 3 consistently supported a 40–50% lower risk of hemorrhoid associated with metformin use in models derived from the unmatched and matched cohorts, respectively; and in either the traditional Cox regression or the Cox regression incorporated with IPTW. The protective effect of metformin was independent of the use of aspirin (models VI and VII) or the use of calcium channel blockers (models VIII and IX).

**TABLE 3:** Sensitivity analyses for estimating hazard ratios for hemorrhoid by metformin exposure.

Model/Metformin use	IncidentCase number	CasesFollowed	Person-years	Incidence rate(per 100,000 person-years)	Traditional Cox model			IPTW model		
					HR	95% CI	p Value	HR	95% CI	p Value
Unmatched cohort										
I. Excluding two consecutive prescriptions of metformin spanning more than 4 months										
Never users	2,025	19,523	80,153.12	2,526.41	1.000			1.000		
Ever users	2,515	52,075	220,925.10	1,138.39	0.437	(0.410–0.466)	<0.0001	0.448	(0.423–0.476)	<0.0001
II. Excluding patients treated with incretin-based therapies during follow-up										
Never users	2,002	18,510	75,573.20	2,649.09	1.000			1.000		
Ever users	7,588	119,286	526,011.63	1,442.55	0.533	(0.506–0.561)	<0.0001	0.540	(0.514–0.567)	<0.0001
III. Patients enrolled during 1999–2002										
Never users	855	8,637	34,586.82	2,472.04	1.000			1.000		
Ever users	5,037	90,236	421,633.37	1,194.64	0.475	(0.440–0.513)	<0.0001	0.473	(0.440–0.508)	<0.0001
IV. Patients enrolled during 2003–2005										
Never users	1,170	10,886	45,566.30	2,567.69	1.000			1.000		
Ever users	3,174	62,111	270,852.70	1,171.85	0.440	(0.409–0.474)	<0.0001	0.455	(0.426–0.487)	<0.0001
V. Defining hemorrhoid as a primary diagnosis at hospitalization										
Never users	349	21,256	90,841.19	384.19	1.000			1.000		
Ever users	1,381	169,495	783,364.48	176.29	0.473	(0.417–0.536)	<0.0001	0.455	(0.405–0.512)	<0.0001
VI. Patients receiving aspirin										
Never users	1,211	11,985	48,552.79	2,494.19	1.000			1.000		
Ever users	5,211	96,528	442,205.39	1,178.41	0.457	(0.428–0.488)	<0.0001	0.464	(0.436–0.494)	<0.0001
VII. Patients not receiving aspirin										
Never users	814	7,538	31,600.33	2,575.92	1.000			1.000		
Ever users	3,000	55,819	250,280.68	1,198.65	0.477	(0.439–0.518)	<0.0001	0.462	(0.428–0.499)	<0.0001
VIII. Patients receiving calcium channel blockers										
Never users	1,315	13,118	53,210.21	2,471.33	1.000			1.000		
Ever users	5,168	94,952	432,049.30	1,196.16	0.469	(0.440–0.501)	<0.0001	0.477	(0.449–0.507)	<0.0001
IX. Patients not receiving calcium channel blockers										
Never users	710	6,405	26,942.91	2,635.20	1.000			1.000		
Ever users	3,043	57,395	260,436.76	1,168.42	0.455	(0.417–0.496)	<0.0001	0.439	(0.405–0.477)	<0.0001
Matched cohort										
I. Excluding two consecutive prescriptions of metformin spanning more than 4 months										
Never users	2,022	19,498	80,068.10	2,525.35	1.000			1.000		
Ever users	349	7,170	30,027.67	1,162.26	0.456	(0.407–0.511)	<0.0001	0.459	(0.410–0.514)	<0.0001
II. Excluding patients treated with incretin-based therapies during follow-up										
Never users	1,999	18,486	75,489.58	2,648.05	1.000			1.000		
Ever users	1,034	15,806	68,477.61	1,509.98	0.567	(0.525–0.611)	<0.0001	0.567	(0.526–0.612)	<0.0001
III. Patients enrolled during 1999–2002										
Never users	854	8,627	34,557.82	2,471.22	1.000			1.000		
Ever users	676	11,583	53,212.25	1,270.38	0.496	(0.448–0.550)	<0.0001	0.509	(0.460–0.563)	<0.0001
IV. Patients enrolled during 2003–2005										
Never users	1,168	10,871	45,510.28	2,566.45	1.000			1.000		
Ever users	413	7,915	33,984.45	1,215.26	0.464	(0.414–0.519)	<0.0001	0.471	(0.421–0.527)	<0.0001
V. Defining hemorrhoid as a primary diagnosis at hospitalization										
Never users	347	21,199	90,667.01	382.72	1.000			1.000		
Ever users	179	21,199	96,460.75	185.57	0.481	(0.401–0.577)	<0.0001	0.484	(0.404–0.579)	<0.0001
VI. Patients receiving aspirin										
Never users	1,208	11,966	48,487.73	2,491.35	1.000			1.000		
Ever users	647	12,019	53,843.20	1,201.64	0.473	(0.429–0.520)	<0.0001	0.479	(0.436–0.527)	<0.0001
VII. Patients not receiving aspirin										
Never users	814	7,532	31,580.38	2,577.55	1.000			1.000		
Ever users	442	7,479	33,353.50	1,325.20	0.510	(0.454–0.573)	<0.0001	0.512	(0.456–0.574)	<0.0001
VIII. Patients receiving calcium channel blockers										
Never users	1,312	13,099	53,141.72	2,468.87	1.000			1.000		
Ever users	721	13,036	58,168.46	1,239.50	0.493	(0.450–0.540)	<0.0001	0.500	(0.456–0.547)	<0.0001
IX. Patients not receiving calcium channel blockers										
Never users	710	6,399	26,926.38	2,636.82	1.000			1.000		
Ever users	368	6,462	29,028.24	1,267.73	0.481	(0.423–0.546)	<0.0001	0.478	(0.422–0.542)	<0.0001

Hemorrhoid was based on a diagnosis made at the out-patient clinics or during hospitalization in all models except Model V.

IPTW, inverse probability of treatment weighting; HR, hazard ratio; CI, confidence interval.

## Discussion

The is the first population-based observational study that showed an overall risk reduction of hemorrhoid associated with metformin use in patients with type 2 diabetes mellitus (Tables 2 and 3, Figures 2–5). A dose-response pattern could be seen in all analyses (Table 2, Figures 3–5).

The mechanisms of a reduced risk of hemorrhoid associated with metformin use require further investigation, but some basic research may provide tentative and reasonable explanations. Results from *in vitro* and *in vivo* studies suggested that metformin may exert cardiac and vascular protective effects via 5′-adenosine monophosphate-activated protein kinase (AMPK)-dependent and AMPK-independent pathways (Nesti and Natali, 2017). Pro-inflammation is a characteristic of insulin resistance (Grandl and Wolfrum, 2018). Metformin increases the expression of insulin receptor and activates tyrosine kinase, and therefore improves insulin resistance (Viollet et al., 2012). Additionally, by changing the composition of the gut microbiota, metformin use is associated with an increase in *Akkermansia* species, which have been shown to improve insulin resistance and reduce tissue inflammation (Hur and Lee, 2015). Metformin may also inhibit the transforming growth factor-beta one signaling pathways (Song et al., 2017), which are activated in cancer cells and several other human diseases involving autoimmunity, fibrosis and cardiovascular system (Serralheiro et al., 2017). Irritable bowel syndrome may cause hemorrhoid (Johannsson et al., 2005; Helvaci et al., 2009) and peptide YY plays an important role in the pathophysiology of irritable bowel syndrome (El-Salhy et al., 2020). Metformin has profound effects on gut hormone signaling including glucagon-like peptide 1 and peptide YY (Glossmann and Lutz, 2019). Whether metformin may prevent the development of hemorrhoid through its actions on gut hormones is an interesting research topic awaiting more in-depth investigation. Metformin may cause increased levels of growth differentiation factor 15; and this increase mediates its effect of body weight loss (Coll et al., 2020). Therefore, metformin may also protect against hemorrhoid by weight reduction following its use. Taken together, metformin may reduce the risk of hemorrhoid via multiple mechanisms by improving insulin resistance, reducing inflammation and fibrosis, affecting gut hormone signaling and weight reduction.

The findings of the present study extended the beneficial effects beyond glycemic control of metformin to the prevention of a very common clinical disease of hemorrhoid. There are some clinical implications. First, metformin has many beneficial effects beyond its glucose lowering effect. These include insulin sensitization, anti-inflammation, cardiovascular protection, anti-aging, anti-cancer and even anti-microbial effects (Maniar et al., 2017; Tseng, 2018a; Tseng, 2018b; Malik et al., 2018). Together with our recent studies, metformin may also exert protection against the development of venous diseases like varicose veins (Tseng, 2020) and hemorrhoid (findings of the present study). These provide good rationales for the use of metformin as the first-line therapeutic drug in the treatment of type 2 diabetes mellitus as recommended by major treatment guidelines (American Diabetes Association, 2017; Salvatore et al., 2020). Second, because all metformin-treated patients seemed to benefit from such a protective effect disregarding the units of DDD taken per day and the protective effect was mainly observed after a cumulative duration of 2 years or a cumulative dose of 750 grams, the use of metformin should be maintained to reach these thresholds when other antidiabetic drugs are added for better glycemic control. Third, the saving of the total healthcare expenditures for the management of other clinical diseases that can be prevented by the continuous use of metformin in the diabetes patients is expected to surpass the drug cost of metformin, an inexpensive drug that does not cause hypoglycemia by itself. Fourth, this observational study gives good rationale for initiating clinical trials to investigate the preventive and therapeutic effects of metformin on hemorrhoid, in either the diabetes patients or the non-diabetes people. However, at this moment, it is still not realistic or justified to recommend metformin for the prevention of hemorrhoid and hemorrhoid-associated complications until the findings are further confirmed by additional observation studies or by clinical trials. If such a protective effect can be confirmed, the clinical usefulness of metformin will be expanded.

In recent years, administrative databases have been popularly used to evaluate long-term safety or beneficial or side effects of medications in pharmacoepidemiological studies. These big data analyses are especially useful for outcomes with low incidence or when randomized trials are not practical. However, some methodological limitations should be carefully addressed. These may include selection bias, prevalent user bias, immortal time bias and confounding by indication.

The present study was designed and conducted to address these potential methodological limitations. First, selection bias can be avoided by using the nationwide NHI database that covers >99% of the population. Second, the potential risk of prevalent user bias was prevented by enrolling patients with new-onset diabetes mellitus and new users of metformin. Additionally, the exclusion of ever users of metformin who had ever been treated with other antidiabetic drugs before metformin was initiated (Figure 1) might have reduced the impacts of other antidiabetic drugs that could occur and be carried over to the period when metformin was initiated.

Third, we tried our best to reduce the immortal time bias. Immortal time refers to the follow-up period during which the outcome cannot happen, and immortal time bias can be introduced when treatment status or follow-up time is inappropriately assigned (Lévesque et al., 2010). We tried to exclude patients with uncertain diagnosis of diabetes mellitus by enrolling only patients who had been prescribed antidiabetic drugs for 2 or more times (Figure 1). Misclassification of treatment status with metformin was not likely because all prescription information was available in the NHI reimbursement database during the long follow-up period. The immortal time between the diagnosis of diabetes mellitus and the initiation of antidiabetic drugs and the immortal time during the short follow-up period of <180 days had been purposely excluded in the calculation of person-years. It is worthy to note that the immortal time pointed out by Lévesque et al. (2010) during the waiting period between drug prescription and dispense at hospital discharge would not happen in Taiwan because all patients can get their discharge drugs from the hospital at the time they are discharged.

Fourth, we aimed at reducing the confounding by indication by using the PS-matched cohort and the Cox regression incorporated with IPTW (Tables 2 and 3). Because all values of standardized difference were <10% in the matched cohort (Table 1), the possibility of residual confounding from the covariates was small in the models created from the matched cohort. Additionally, the consistency of the findings in the unmatched cohort, in the analyses by using the traditional Cox regression and in the sensitivity analyses all supported that the results are robust and not liable to changes in different cohorts or by using different statistical methods.

Study limitations may include a lack of measurement data of confounders like biochemical and humoral profiles, anthropometric factors, lifestyle, physical activity, history of constipation and diarrhea, history of sexual intercourse, daily standing time, numbers of births and pelvic disease in women, cigarette smoking, alcohol drinking, dietary pattern, family history and genetic parameters. Visceral neuropathy or pudendal neuralgia may cause chronic constipation and hemorrhoid (Bharucha et al., 2013). However, we do not have such information in the database for analyses. Because the diagnosis of hemorrhoid was based on ICD-9-CM code without supportive laboratory data, it is possible that misclassification of hemorrhoid could not be entirely excluded. However, the hazard ratios would only be underestimated if the misclassifications were not differential in ever users and never users of metformin (Kesmodel, 2018). To further confirm the preventive role of metformin on hemorrhoid, sensitivity analyses were conducted by re-defining the outcome with a more stringent criterion of a primary diagnosis of hemorrhoid during hospitalization (Models V, Table 3). The estimated hazard ratios were very similar to those derived from the main analyses in Table 2. Finally, there is a possibility that some patients might not report their symptoms or signs related to hemorrhoid to their attending doctors and might have bought medications to treat their hemorrhoids by themselves. Because the coverage rate of NHI is very high and the patients always do not need to give extra payment if they get their medications for hemorrhoid at the same time when they receive their antidiabetic prescriptions, it is believed that the diabetes patients would rather report related symptoms to their doctors and requested medications for their hemorrhoids than buy over-the-counter medications by themselves that would cost extra expenses out of their pockets. Furthermore, if such misclassification was not differential between ever users and never users of metformin, the estimated hazard ratios would only be biased toward the null (Kesmodel, 2018).

There are some additional strengths. First, self-reporting bias could be much reduced by using medical records. Second, although detection bias related to different socioeconomic status can be a problem in some countries, this was less likely a problem here because the drug cost-sharing in the Taiwan’s NHI healthcare system is low. Furthermore, much expense can be waived in veterans and in patients with low-income or when the patients receive prescription refills for chronic disease.

## Conclusion

The findings of this study support a lower risk of hemorrhoid associated with chronic therapy of metformin in patients with type 2 diabetes mellitus in Taiwan when the cumulative duration is >2 years or the cumulative dose is >750 grams. However, confirmation in other populations is necessary. Because metformin does not cause hypoglycemia when used as a monotherapy and it is inexpensive and safe for long-term use, its protective effect on hemorrhoid is worthy of more investigation, not only in patients with diabetes mellitus but also in non-diabetes people.

## Data Availability

The datasets for this article are not publicly available because public availability of the dataset is restricted by local regulations to protect privacy.
